# Supplementary material for: A naturalistic study of brushing patterns using powered toothbrushes
Source: PLoS One. 2022 May 19;17(5):e0263638. doi: 10.1371/journal.pone.0263638 (PMC9119504; doi:10.1371/journal.pone.0263638)
Supplement: S1 Appendix — (DOCX) [file pone.0263638.s001.docx]

Supplementary Information

# A1. Brushing duration of each dental surface

## A1.1. Modeling

Several participants skipped some regions altogether in some or all brushing sessions. Therefore, the brushing durations of any dental surface was often equal to zero, a statistical phenomenon referred to as zero-inflation. Zero-inflation for brushing duration distribution of MaxRO is shown in Fig A1.1.1.


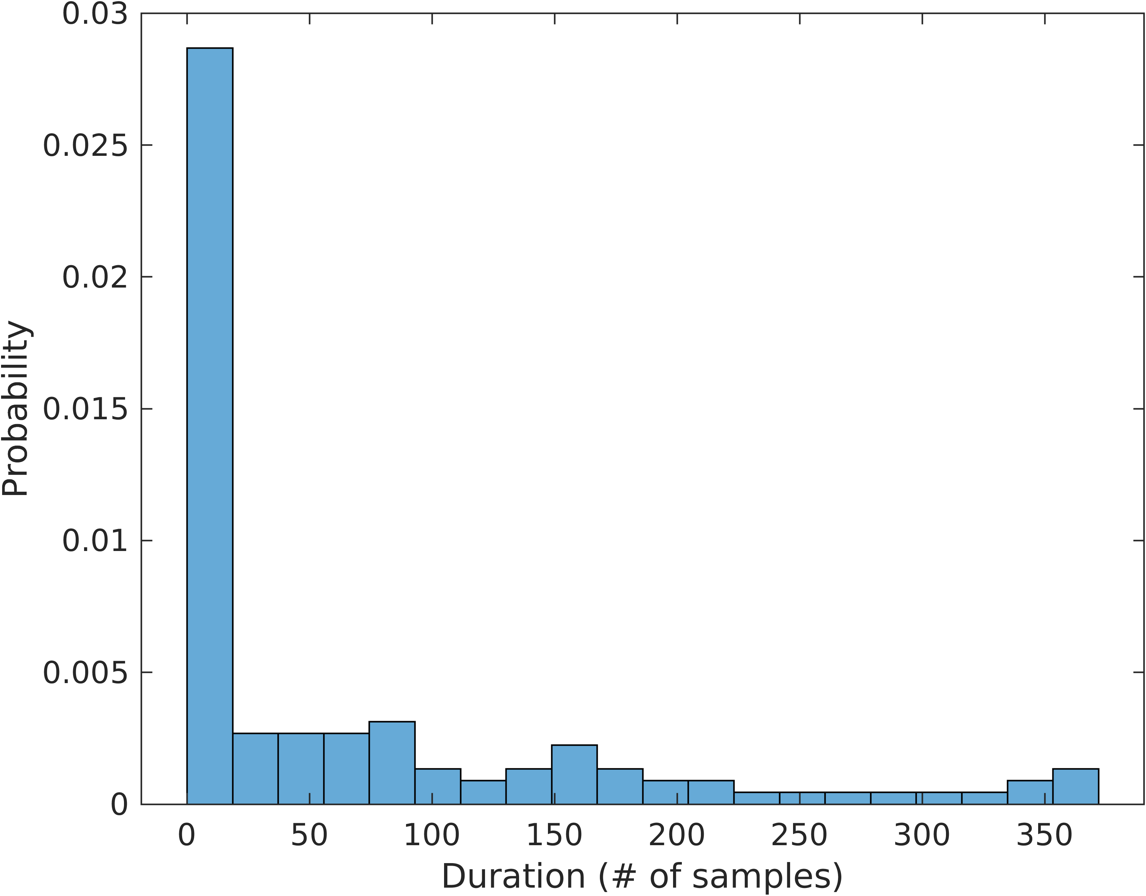


**Fig A1.1.1. Zero-inflated distribution of brushing duration on MaxRO.**

As a result, we fit a zero-inflated negative binomial regression model for the amount of time (measured in counts of 25 Hz samples) spent brushing each dental surface, with a log-link and a negative binomial outcome distribution for the count submodel and a logistic link and a Bernoulli outcome distribution for the zero-inflation submodel. Both submodels had fixed effects for tooth surface, mouth side, and jaw, and random effects on the intercept by session nested in participant, to account for participant-to-participant and session-to-session differences in overall brushing duration, and participant-specific overdispersion parameters to account for participant-to-participant differences in residual variance. The count submodel also included participant-specific random effects for tooth surface, mouth side, and jaw. We attempted to add participant-specific random effects for tooth surface, mouth side, and jaw in the zero-inflation submodel, but the estimation algorithm failed to converge for that extended model.

The brushing duration $Y_{ijk}$ of participant $i\in\left\{ 1:12 \right\}$, in session $j\in1:10$, of dental surface $k\in$ {MaxRO, MaxRB, MaxAB, MaxLB, MaxLO, MaxRL, MaxAL, MaxLL, ManRO, ManRB, ManAB, ManLB, ManLO, ManRL, ManAL, ManLL} is modeled as follows:

$$Z_{ijk}\sim\mathrm{Bernoulli}\left( \pi_{ijk} \right)$$

$$Y_{ijk}\sim\left\{ \begin{matrix} 0 & \mathrm{if}Z_{ijk}=1 \\ \mathrm{NB}\left( \lambda_{ijk},\kappa_{i} \right) & \mathrm{if} Z_{ijk}=0 \end{matrix} \right.$$

We used the following parameterization of the negative binomial distribution:

$$p\left( Y_{ijk}=y | Z_{ijk}=0 \right)=\frac{\left( \lambda_{ijk} \right)^{y}}{y!}\cdot\frac{\Gamma\left( y+\kappa_{i} \right)}{\Gamma\left( \kappa_{i} \right)\left( \kappa_{i}+\lambda_{ijk} \right)^{y}}\cdot\left( 1+\frac{\lambda_{ijk}}{\kappa_{i}} \right)^{\kappa_{i}}$$

Hence:

$$\mathbb{E}\left( Y_{ijk} | Z_{ijk}=0 \right)=\lambda_{ijk}$$

$$\mathrm{Var} \left( Y_{ijk} | Z_{ijk}=0 \right)=\lambda_{ijk}\left( 1+\frac{\lambda_{ijk}}{\kappa_{i}} \right)$$

It should be noted that larger values of $\kappa_{i}$ correspond to smaller variances.

We modeled $\lambda_{ijk}=\mathbb{E}\left( Y_{ijk} | Z_{ijk}=0 \right)$ as follows:

$$\log\left( \lambda_{ijk} \right)=\beta_{0ij}+\beta_{\mathcal{M}i}1\left\{ k\mathcal{\in M} \right\}+\beta_{Oi}1\left\{ k\in\mathcal{O} \right\}+ \beta_{\mathcal{L}i}1\left\{ k\mathcal{\in L} \right\}+ \beta_{\mathcal{A}i}1\left\{ k\in\mathcal{A} \right\}+ \beta_{\mathcal{G}i}1\left\{ k\in\mathcal{G} \right\}$$

$$\beta_{0ij}=\beta_{0}+\alpha_{0i}+\gamma_{ij}$$

$$\beta_{\mathcal{M}i}=\beta_{\mathcal{M}}+\alpha_{\mathcal{M}i}$$

$$\gamma_{ij}\sim N\left( 0,\sigma_{\gamma}^{2} \right)$$

$$\left( \begin{matrix} \alpha_{0i} \\ \alpha_{\mathcal{M}i} \\ \vdots\end{matrix} \right)\sim N\left( \boldsymbol{0},\left[ \begin{matrix} \sigma_{\alpha_{0}}^{2} & \sigma_{\alpha_{0}}\sigma_{\mathcal{M}}\rho_{\alpha_{0}\mathcal{M}} & \cdots\\ \sigma_{\alpha_{0}}\sigma_{\mathcal{M}}\rho_{\alpha_{0}\mathcal{M}} & \sigma_{\mathcal{M}}^{2} & \ddots\\ \vdots& \ddots& \ddots\end{matrix} \right] \right)$$

$$\log(\kappa_{i})=\eta_{i}$$

$$\mathrm{logit} \left( \pi_{ijk} \right)=\theta_{0ij}+\theta_{\mathcal{M}}1\left\{ k\mathcal{\in M} \right\}+\theta_{\mathcal{O}}1\left\{ k\in\mathcal{O} \right\}+ \theta_{\mathcal{L}}1\left\{ k\mathcal{\in L} \right\}+ \theta_{\mathcal{A}}1\left\{ k\in\mathcal{A} \right\}+\theta_{\mathcal{G}}1\left\{ k\in\mathcal{G} \right\}$$

$$\theta_{0ij}=\theta_{0}+\delta_{i}+\epsilon_{ij}$$

$$\delta_{i}\sim N\left( 0,\sigma_{\delta}^{2} \right)$$

$$\epsilon_{ij}\sim N\left( 0,\sigma_{\epsilon}^{2} \right)$$

In the preceding expressions, $\mathcal{M}\mathcal{, O, L,}\mathcal{A, G}$ denote the sets of maxillary, occlusal, lingual, anterior, and gauche (left) regions, respectively; e.g., $\mathcal{M=}$ {MaxRO, MaxRB, MaxAB, MaxLB, MaxLO, MaxRL, MaxAL, MaxLL} and $\mathcal{O=}$ {MaxRO, ManRO, MaxLO, ManLO}.

## A1.2. Parameter Estimates

The AIC for this model was 18,598.76, which was 69,183.57 less than a zero-inflated Poisson model with the same fixed and random effects. The BIC for this model was 18,865.64, which was 69,116.85 less than the zero-inflated Poisson model with the same fixed and random effects.

Tables below summarizes the estimated parameters for the count and zero-inflated submodels.

**Table A1.2.1 Estimated fixed effects of count submodel for brushing duration of each dental surface.**

| Parameter | Log-Mean | SE | 95% CI | p |
| --- | --- | --- | --- | --- |
| $\beta_{0}$: Intercept | 5.33 | 0.11 | (5.12, 5.54) | < .001 |
| $\beta_{\mathcal{L}}$: Surface (Lingual) | -0.78 | 0.22 | (-1.21, -0.35) | < .001 |
| $\beta_{\mathcal{O}}$: Surface (Occlusal) | -0.80 | 0.16 | (-1.13, -0.48) | < .001 |
| $\beta_{\mathcal{A}}$: Side (Anterior) | -0.01 | 0.09 | (-0.19, 0.17) | 0.892 |
| $\beta_{\mathcal{G}}$: Side (Left) | -3.29e-03 | 0.08 | (-0.15, 0.15) | 0.966 |
| $\beta_{\mathcal{M}}$: Jaw (Maxillary) | 0.09 | 0.14 | (-0.18, 0.37) | 0.508 |

**Table A1.2.2. Estimated fixed effects of zero-inflation submodel for brushing duration of each dental surface.**

| Parameter | Log-Odds | SE | 95% CI | p |
| --- | --- | --- | --- | --- |
| $\theta_{0}$: (Intercept) | -4.25 | 0.43 | (-5.09, -3.41) | < .001 |
| $\theta_{\mathcal{L}}$: Surface (Lingual) | 3.04 | 0.23 | (2.59, 3.49) | < .001 |
| $\theta_{\mathcal{O}}$: Surface (Occlusal) | 3.47 | 0.25 | (2.98, 3.96) | < .001 |
| $\theta_{\mathcal{A}}$: Side (Anterior) | 0.17 | 0.19 | (-0.20, 0.53) | 0.363 |
| $\theta_{\mathcal{G}}$: Side (Left) | 0.13 | 0.14 | (-0.16, 0.41) | 0.381 |
| $\theta_{\mathcal{M}}$: Jaw (Maxillary) | 0.68 | 0.13 | (0.42, 0.94) | < .001 |

**Table A1.2.3. Estimated participant-specific overdispersion parameters of count submodel for brushing duration of each dental surface.**

| Parameter | Coefficient | SE | 95% CI | p |
| --- | --- | --- | --- | --- |
| $\eta_{1}$: Participant 1 | 1.23 | 0.12 | (1.00, 1.46) | < .001 |
| $\eta_{2}$: Participant 2 | 1.55 | 0.14 | (1.29, 1.82) | < .001 |
| $\eta_{3}$: Participant 3 | 1.16 | 0.13 | (0.91, 1.40) | < .001 |
| $\eta_{4}$: Participant 4 | 0.84 | 0.14 | (0.58, 1.11) | < .001 |
| $\eta_{5}$: Participant 5 | 0.64 | 0.13 | (0.38, 0.90) | < .001 |
| $\eta_{6}$: Participant 6 | 0.74 | 0.16 | (0.43, 1.05) | < .001 |
| $\eta_{7}$: Participant 7 | 1.05 | 0.19 | (0.68, 1.43) | < .001 |
| $\eta_{8}$: Participant 8 | 0.77 | 0.12 | (0.54, 1.00) | < .001 |
| $\eta_{9}$: Participant 9 | 0.56 | 0.12 | (0.31, 0.80) | < .001 |
| $\eta_{10}$: Participant 10 | 0.98 | 0.11 | (0.75, 1.20) | < .001 |
| $\eta_{11}$: Participant 11 | 0.72 | 0.14 | (0.44, 0.99) | < .001 |
| $\eta_{12}$: Participant 12 | 0.94 | 0.12 | (0.70, 1.18) | < .001 |

**Table A1.2.4. Estimated standard deviations of random effects of count submodel for brushing duration of each dental surface.**

| Parameter | Coefficient |
| --- | --- |
| $\sigma_{\alpha_{0}}$: SD (Intercept: Participant) | 0.34 |
| $\sigma_{\gamma}$: SD (Intercept: Participant:Session) | 9.56e-05 |
| $\sigma_{\mathcal{L}}$: SD (SurfaceLingual: Participant) | 0.68 |
| $\sigma_{\mathcal{O}}$: SD (SurfaceOcclusal: Participant) | 0.54 |
| $\sigma_{\mathcal{M}}$: SD (JawMaxillary: Participant) | 0.47 |
| $\sigma_{\mathcal{A}}$: SD (SideAnterior: Participant) | 0.26 |
| $\sigma_{\mathcal{G}}$: SD (SideLeft: Participant) | 0.22 |

**Table A1.2.5. Estimated standard deviations of random effects of zero-inflated submodel for brushing duration of each dental surface.**

| Parameter | Coefficient |
| --- | --- |
| $\sigma_{\epsilon}$: SD (Intercept: <Session:Participant>) | 0.12 |
| $\sigma_{\delta}$: SD (Intercept: Participant) | 1.20 |

**Table A1.2.6. Estimated participant-level random effects of count submodel for brushing duration of each dental surface.**

| Participant # | Parameter | Estimate | Std. Error | Pr(>\|z\|) | 2.5 % | 97.5 % |
| --- | --- | --- | --- | --- | --- | --- |
| 1 | $\alpha_{01}$ : (Intercept) | 0.132 | 0.141 | 0.347 | -0.143 | 0.408 |
| 2 | $\alpha_{02}$ : (Intercept) | 0.363 | 0.140 | 0.010 | 0.087 | 0.638 |
| 3 | $\alpha_{03}$ : (Intercept) | -0.008 | 0.152 | 0.958 | -0.306 | 0.290 |
| 4 | $\alpha_{04}$ : (Intercept) | 0.506 | 0.167 | 0.003 | 0.178 | 0.834 |
| 5 | $\alpha_{05}$ : (Intercept) | -0.305 | 0.181 | 0.091 | -0.660 | 0.049 |
| 6 | $\alpha_{06}$ : (Intercept) | 0.091 | 0.166 | 0.584 | -0.234 | 0.416 |
| 7 | $\alpha_{07}$ : (Intercept) | -0.225 | 0.206 | 0.275 | -0.629 | 0.179 |
| 8 | $\alpha_{08}$ : (Intercept) | -0.273 | 0.162 | 0.092 | -0.589 | 0.044 |
| 9 | $\alpha_{09}$ : (Intercept) | -0.473 | 0.177 | 0.007 | -0.820 | -0.127 |
| 10 | $\alpha_{010}$ : (Intercept) | -0.114 | 0.155 | 0.459 | -0.417 | 0.188 |
| 11 | $\alpha_{011}$ : (Intercept) | 0.496 | 0.185 | 0.007 | 0.134 | 0.858 |
| 12 | $\alpha_{012}$ : (Intercept) | -0.267 | 0.158 | 0.091 | -0.575 | 0.042 |
| 1 | $\alpha_{\mathcal{L}1}$ : SurfaceLingual | 0.188 | 0.239 | 0.431 | -0.280 | 0.657 |
| 2 | $\alpha_{\mathcal{L}2}$ : SurfaceLingual | 0.498 | 0.236 | 0.034 | 0.037 | 0.960 |
| 3 | $\alpha_{\mathcal{L}3}$ : SurfaceLingual | 0.401 | 0.240 | 0.095 | -0.070 | 0.872 |
| 4 | $\alpha_{\mathcal{L}4}$ : SurfaceLingual | -0.593 | 0.264 | 0.025 | -1.110 | -0.076 |
| 5 | $\alpha_{\mathcal{L}5}$ : SurfaceLingual | -0.276 | 0.257 | 0.282 | -0.779 | 0.227 |
| 6 | $\alpha_{\mathcal{L}6}$ : SurfaceLingual | -0.794 | 0.425 | 0.061 | -1.627 | 0.038 |
| 7 | $\alpha_{\mathcal{L}7}$ : SurfaceLingual | -0.951 | 0.869 | 0.274 | -2.654 | 0.752 |
| 8 | $\alpha_{\mathcal{L}8}$ : SurfaceLingual | 0.547 | 0.251 | 0.029 | 0.055 | 1.039 |
| 9 | $\alpha_{\mathcal{L}9}$ : SurfaceLingual | 0.940 | 0.262 | <0.001 | 0.426 | 1.453 |
| 10 | $\alpha_{\mathcal{L}10}$ : SurfaceLingual | 0.496 | 0.245 | 0.043 | 0.016 | 0.975 |
| 11 | $\alpha_{\mathcal{L}11}$ : SurfaceLingual | -1.015 | 0.304 | 0.001 | -1.610 | -0.420 |
| 12 | $\alpha_{\mathcal{L}12}$ : SurfaceLingual | 0.504 | 0.249 | 0.043 | 0.015 | 0.992 |
| 1 | $\alpha_{\mathcal{O}1}$ : SurfaceOcclusal | 0.312 | 0.194 | 0.108 | -0.069 | 0.693 |
| 2 | $\alpha_{\mathcal{O}2}$ : SurfaceOcclusal | 0.091 | 0.225 | 0.686 | -0.350 | 0.533 |
| 3 | $\alpha_{\mathcal{O}3}$ : SurfaceOcclusal | -0.996 | 0.257 | <0.001 | -1.499 | -0.492 |
| 4 | $\alpha_{\mathcal{O}4}$ : SurfaceOcclusal | -0.545 | 0.253 | 0.032 | -1.042 | -0.048 |
| 5 | $\alpha_{\mathcal{O}5}$ : SurfaceOcclusal | -0.824 | 0.259 | 0.001 | -1.332 | -0.315 |
| 6 | $\alpha_{\mathcal{O}6}$ : SurfaceOcclusal | 0.567 | 0.218 | 0.009 | 0.140 | 0.994 |
| 7 | $\alpha_{\mathcal{O}7}$ : SurfaceOcclusal | 0.109 | 0.297 | 0.714 | -0.474 | 0.692 |
| 8 | $\alpha_{\mathcal{O}8}$ : SurfaceOcclusal | 0.390 | 0.214 | 0.068 | -0.029 | 0.809 |
| 9 | $\alpha_{\mathcal{O}9}$ : SurfaceOcclusal | -0.311 | 0.259 | 0.229 | -0.819 | 0.196 |
| 10 | $\alpha_{\mathcal{O}10}$ : SurfaceOcclusal | 0.569 | 0.208 | 0.006 | 0.161 | 0.977 |
| 11 | $\alpha_{\mathcal{O}11}$ : SurfaceOcclusal | 0.301 | 0.236 | 0.201 | -0.161 | 0.764 |
| 12 | $\alpha_{\mathcal{O}12}$ : SurfaceOcclusal | 0.266 | 0.209 | 0.204 | -0.144 | 0.677 |
| 1 | $\alpha_{\mathcal{M}1}$ : JawMaxillary | -0.148 | 0.164 | 0.365 | -0.469 | 0.172 |
| 2 | $\alpha_{\mathcal{M}2}$ : JawMaxillary | -0.046 | 0.164 | 0.781 | -0.366 | 0.275 |
| 3 | $\alpha_{\mathcal{M}3}$ : JawMaxillary | -0.084 | 0.168 | 0.615 | -0.414 | 0.245 |
| 4 | $\alpha_{\mathcal{M}4}$ : JawMaxillary | -0.340 | 0.193 | 0.079 | -0.719 | 0.039 |
| 5 | $\alpha_{\mathcal{M}5}$ : JawMaxillary | -0.476 | 0.193 | 0.014 | -0.854 | -0.097 |
| 6 | $\alpha_{\mathcal{M}6}$ : JawMaxillary | 0.190 | 0.195 | 0.330 | -0.192 | 0.573 |
| 7 | $\alpha_{\mathcal{M}7}$ : JawMaxillary | 1.404 | 0.236 | <0.001 | 0.941 | 1.866 |
| 8 | $\alpha_{\mathcal{M}8}$ : JawMaxillary | -0.033 | 0.177 | 0.850 | -0.380 | 0.313 |
| 9 | $\alpha_{\mathcal{M}9}$ : JawMaxillary | 0.001 | 0.198 | 0.996 | -0.387 | 0.389 |
| 10 | $\alpha_{\mathcal{M}10}$ : JawMaxillary | -0.097 | 0.172 | 0.571 | -0.434 | 0.240 |
| 11 | $\alpha_{\mathcal{M}11}$ : JawMaxillary | -0.264 | 0.212 | 0.212 | -0.679 | 0.150 |
| 12 | $\alpha_{\mathcal{M}12}$ : JawMaxillary | -0.107 | 0.177 | 0.544 | -0.453 | 0.239 |
| 1 | $\alpha_{\mathcal{A}1}$ : SideAnterior | 0.177 | 0.139 | 0.203 | -0.095 | 0.448 |
| 2 | $\alpha_{\mathcal{A}2}$ : SideAnterior | -0.031 | 0.130 | 0.813 | -0.285 | 0.223 |
| 3 | $\alpha_{\mathcal{A}3}$ : SideAnterior | -0.009 | 0.143 | 0.951 | -0.289 | 0.271 |
| 4 | $\alpha_{\mathcal{A}4}$ : SideAnterior | 0.020 | 0.162 | 0.901 | -0.297 | 0.338 |
| 5 | $\alpha_{\mathcal{A}5}$ : SideAnterior | 0.335 | 0.186 | 0.072 | -0.030 | 0.699 |
| 6 | $\alpha_{\mathcal{A}6}$ : SideAnterior | -0.426 | 0.271 | 0.116 | -0.958 | 0.105 |
| 7 | $\alpha_{\mathcal{A}7}$ : SideAnterior | -0.464 | 0.230 | 0.043 | -0.914 | -0.014 |
| 8 | $\alpha_{\mathcal{A}8}$ : SideAnterior | 0.024 | 0.148 | 0.872 | -0.265 | 0.313 |
| 9 | $\alpha_{\mathcal{A}9}$ : SideAnterior | 0.088 | 0.168 | 0.600 | -0.241 | 0.418 |
| 10 | $\alpha_{\mathcal{A}10}$ : SideAnterior | 0.236 | 0.151 | 0.118 | -0.060 | 0.533 |
| 11 | $\alpha_{\mathcal{A}11}$ : SideAnterior | -0.116 | 0.184 | 0.529 | -0.477 | 0.245 |
| 12 | $\alpha_{\mathcal{A}12}$ : SideAnterior | 0.170 | 0.148 | 0.248 | -0.119 | 0.460 |
| 1 | $\alpha_{\mathcal{G}1}$ : SideLeft | -0.009 | 0.112 | 0.934 | -0.229 | 0.210 |
| 2 | $\alpha_{\mathcal{G}2}$ : SideLeft | -0.204 | 0.120 | 0.090 | -0.439 | 0.032 |
| 3 | $\alpha_{\mathcal{G}3}$ : SideLeft | 0.086 | 0.125 | 0.489 | -0.159 | 0.331 |
| 4 | $\alpha_{\mathcal{G}4}$ : SideLeft | -0.034 | 0.149 | 0.817 | -0.326 | 0.257 |
| 5 | $\alpha_{\mathcal{G}5}$ : SideLeft | 0.293 | 0.158 | 0.065 | -0.018 | 0.603 |
| 6 | $\alpha_{\mathcal{G}6}$ : SideLeft | -0.074 | 0.154 | 0.631 | -0.375 | 0.227 |
| 7 | $\alpha_{\mathcal{G}7}$ : SideLeft | -0.313 | 0.200 | 0.118 | -0.705 | 0.080 |
| 8 | $\alpha_{\mathcal{G}8}$ : SideLeft | 0.091 | 0.122 | 0.458 | -0.148 | 0.329 |
| 9 | $\alpha_{\mathcal{G}9}$ : SideLeft | 0.290 | 0.134 | 0.030 | 0.028 | 0.552 |
| 10 | $\alpha_{\mathcal{G}10}$ : SideLeft | -0.025 | 0.120 | 0.836 | -0.261 | 0.211 |
| 11 | $\alpha_{\mathcal{G}11}$ : SideLeft | -0.321 | 0.171 | 0.061 | -0.657 | 0.015 |
| 12 | $\alpha_{\mathcal{G}12}$ : SideLeft | 0.249 | 0.137 | 0.068 | -0.019 | 0.518 |

Table A1.2.7. Estimated participant-level random effects of zero-inflated submodel for brushing duration of each dental surface

| Participant # | Parameter | Estimate | Std. Error | Pr(>\|z\|) | 2.5 % | 97.5 % |
| --- | --- | --- | --- | --- | --- | --- |
| 1 | $\delta_{1}$ : (Intercept) | -1.467 | 0.449 | 0.001 | -2.347 | -0.587 |
| 2 | $\delta_{2}$ : (Intercept) | 0.430 | 0.396 | 0.278 | -0.347 | 1.207 |
| 3 | $\delta_{3}$ : (Intercept) | -0.306 | 0.405 | 0.450 | -1.101 | 0.488 |
| 4 | $\delta_{4}$ : (Intercept) | 0.541 | 0.396 | 0.172 | -0.235 | 1.318 |
| 5 | $\delta_{5}$ : (Intercept) | 0.151 | 0.399 | 0.704 | -0.631 | 0.933 |
| 6 | $\delta_{6}$ : (Intercept) | 1.162 | 0.397 | 0.003 | 0.385 | 1.939 |
| 7 | $\delta_{7}$ : (Intercept) | 2.496 | 0.414 | <0.001 | 1.684 | 3.308 |
| 8 | $\delta_{8}$ : (Intercept) | -1.389 | 0.445 | 0.002 | -2.261 | -0.516 |
| 9 | $\delta_{9}$ : (Intercept) | -0.091 | 0.402 | 0.821 | -0.879 | 0.697 |
| 10 | $\delta_{10}$ : (Intercept) | -1.765 | 0.471 | <0.001 | -2.688 | -0.842 |
| 11 | $\delta_{11}$ : (Intercept) | 0.796 | 0.396 | 0.044 | 0.021 | 1.572 |
| 12 | $\delta_{12}$ : (Intercept) | -0.399 | 0.407 | 0.327 | -1.197 | 0.399 |

From the ratio of the estimated standard deviations for participant and session level random effects on the intercept, $\frac{\hat{\sigma}_{\gamma}}{\hat{\sigma}_{\alpha_{0}}}=\frac{9.56e-05}{0.34}<0.1\%$ and $\frac{\hat{\sigma}_{\epsilon}}{\hat{\sigma}_{\delta}}=\frac{0.12}{1.20}=10\%$, we can see that within-participant variability (session-to-session) is much smaller than between-participant variability, on the log-mean scale.

We summarized coefficient of variation of all 16 dental regions of all 12 participants in Table A1.2.8 and illustrated in Fig A1.2.1.


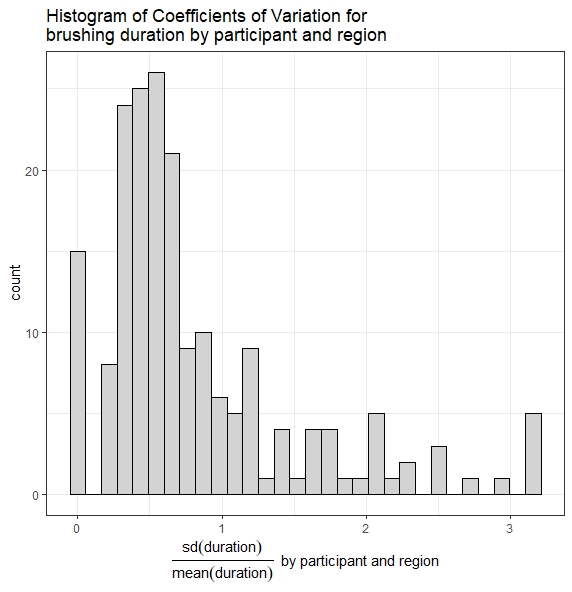


Fig A1.2.1. Coefficient of variation of brushing duration of all regions brushed by all participants

Table A1.2.8 Coefficient of variation of all 16 dental regions for all participants

| Participant | ManAB | ManAL | ManLB | ManLL | ManLO | ManRB | ManRL | ManRO | MaxAB | MaxAL | MaxLB | MaxLL | MaxLO | MaxRB | MaxRL | MaxRO |
| --- | --- | --- | --- | --- | --- | --- | --- | --- | --- | --- | --- | --- | --- | --- | --- | --- |
| 1 | 1.072 | 0.328 | 0.399 | 0.503 | 0.443 | 0.291 | 0.735 | 0.355 | 0.465 | 0.575 | 0.377 | 0.714 | 0.583 | 0.384 | 1.001 | 0.518 |
| 2 | 0.468 | 0.437 | 0.482 | 0.456 | 1.436 | 0.880 | 0.622 | 2.894 | 0.383 | 0.770 | 0.370 | 1.795 | 3.162 | 0.449 | 0.611 | 2.500 |
| 3 | 0.275 | 0.562 | 0.242 | 0.500 | 2.111 | 0.300 | 0.346 | 2.539 | 0.254 | 0.579 | 0.302 | 0.583 | 1.917 | 0.441 | 0.261 | 1.807 |
| 4 | 0.337 | 0.554 | 0.284 | 0.663 | 2.037 | 0.391 | 0.845 | 1.250 | 0.409 | 1.457 | 0.493 | 2.110 | 2.742 | 0.310 | 3.162 | 0 |
| 5 | 0.686 | 1.153 | 0.669 | 1.104 | 1.240 | 0.813 | 1.026 | 0 | 0.326 | 1.224 | 0.891 | 0.994 | 1.476 | 0.995 | 1.091 | 0.835 |
| 6 | 1.158 | 2.108 | 0.830 | 0 | 0.610 | 0.399 | 0 | 1.302 | 0.495 | 3.162 | 0.907 | 0 | 0.639 | 0.718 | 3.162 | 0.376 |
| 7 | 2.250 | 0 | 0.721 | 0 | 0 | 1.192 | 3.162 | 0.688 | 0.397 | 0 | 0.214 | 0 | 0 | 0.249 | 0 | 0 |
| 8 | 0.981 | 0.473 | 0.567 | 0.403 | 0.568 | 0.552 | 0.580 | 0.699 | 0.616 | 0.554 | 0.394 | 0.573 | 1.639 | 0.667 | 0.645 | 0.912 |
| 9 | 0.839 | 0.860 | 0.400 | 0.382 | 1.673 | 0.635 | 0.414 | 1.369 | 0.336 | 1.753 | 0.600 | 0.636 | 2.279 | 0.544 | 0.496 | 1.417 |
| 10 | 0.544 | 0.394 | 0.474 | 0.263 | 0.680 | 0.473 | 0.266 | 0.622 | 0.204 | 0.593 | 0.300 | 1.203 | 0.508 | 0.663 | 0.745 | 0.543 |
| 11 | 0.373 | 2.482 | 0.460 | 1.656 | 0.506 | 0.524 | 1.629 | 1.203 | 0.278 | 2.061 | 0.518 | 0 | 2.182 | 0.519 | 0 | 1.181 |
| 12 | 0.610 | 0.286 | 0.609 | 0.470 | 0.751 | 0.710 | 0.671 | 0.979 | 0.370 | 1.093 | 0.330 | 1.758 | 0.911 | 0.466 | 1.7623 | 1.050 |

As it can be seen all the dental regions which are brushed at all, have a coefficient of variation of more than 20%.

# A2. Duration of excessive brushing pressure on each dental surface

## A2.1. Modeling

As in Section S1, we fit a zero-inflated negative binomial model; both the zero-inflation and count submodels include fixed effects for tooth surface, side, and jaw, and random effects on the intercept by participant and session. Extended models adding participant-level random effects on tooth surface, side and jaw and participant-specific overdispersion parameters failed to converge.

$$Z_{ijk}\sim Bernoulli(\pi_{ijk})$$

$$Y_{ijk}\sim\left\{ \begin{matrix} 0, & Z_{ijk}=1 \\ NB\left( \lambda_{ijk},\kappa\right) & Z_{ijk}=0 \end{matrix} \right.$$

Hence:

$$\lambda_{ijk}\mathbb{=E}\left( Y_{ijk} | Z_{ijk}=0 \right)$$

$$\mathrm{Var} \left( Y_{ijk} | Z_{ijk}=0 \right)=\lambda_{ijk}\left( 1+\frac{\lambda_{ijk}}{\kappa} \right)$$

$\lambda_{ijk}$ is modeled as:

$$\log\left( \lambda_{ijk} \right)=\beta_{0ij}+\beta_{\mathcal{M}}1\left\{ k\mathcal{\in M} \right\}+\beta_{\mathcal{O}}1\left\{ k\in\mathcal{O} \right\}+ \beta_{\mathcal{L}}1\left\{ k\mathcal{\in L} \right\}+ \beta_{\mathcal{A}}1\left\{ k\in\mathcal{A} \right\}+ \beta_{\mathcal{G}}1\left\{ k\in\mathcal{G} \right\}$$

$$\beta_{0ij}=\beta_{0}+\alpha_{0i}+\gamma_{ij}$$

$$\gamma_{ij}\sim N\left( 0,\sigma_{\gamma}^{2} \right)$$

$$\alpha_{0i}\sim N\left( 0,\sigma_{\alpha_{0}}^{2} \right)$$

$$\log(\kappa)=\eta$$

$$\mathrm{logit} \left( \pi_{ijk} \right)=\theta_{0ij}+\theta_{\mathcal{M}}1\left\{ k\mathcal{\in M} \right\}+\theta_{\mathcal{O}}1\left\{ k\in\mathcal{O} \right\}+ \theta_{\mathcal{L}}1\left\{ k\mathcal{\in L} \right\}+ \theta_{\mathcal{A}}1\left\{ k\in\mathcal{A} \right\}+\theta_{\mathcal{G}}1\left\{ k\in\mathcal{G} \right\}$$

$$\theta_{0ij}=\theta_{0}+\delta_{i}+\epsilon_{ij}$$

$$\delta_{i}\sim N\left( 0,\sigma_{\delta}^{2} \right)$$

$$\epsilon_{ij}\sim N\left( 0,\sigma_{\epsilon}^{2} \right)$$

The AIC for this model was 1,252.2, which was 274.6 less than a zero-inflated Poisson model with the same fixed and random effects. The BIC for this model was 1,346.7, which was 269.0 less than the zero-inflated Poisson model with the same fixed and random effects.

## A2.2. Parameter Estimates

Tables below summarizes the estimated parameters for the count and zero-inflated submodels for excessive brushing pressure duration.

**Table A2.2.1. Estimated fixed effects of count submodel for excessive brushing pressure duration on each dental surface.**

| Parameter | Log-Mean | SE | 95% CI | p |
| --- | --- | --- | --- | --- |
| $\beta_{0}$ : (Intercept) | 2.12 | 0.30 | (1.53, 2.71) | < .001 |
| $\beta_{\mathcal{L}}$ : Surface (Lingual) | 0.01 | 0.29 | (-0.56, 0.58) | 0.971 |
| $\beta_{\mathcal{O}}$: Surface (Occlusal) | 0.54 | 0.22 | (0.10, 0.98) | 0.015 |
| $\beta_{\mathcal{A}}$ : Side (Anterior) | 0.04 | 0.32 | (-0.58, 0.66) | 0.889 |
| $\beta_{\mathcal{G}}$ : Side (Left) | -0.04 | 0.20 | (-0.44, 0.37) | 0.862 |
| $\beta_{\mathcal{M}}$ : Jaw (Maxillary) | -0.10 | 0.21 | (-0.51, 0.30) | 0.612 |

**Table A2.2.2. Estimated fixed effects of zero-inflation submodel for excessive brushing pressure duration on each dental surface.**

| Parameter | Log-Odds | SE | 95% CI | p |
| --- | --- | --- | --- | --- |
| $\theta_{0}$ : (Intercept) | 4.87 | 0.93 | (3.04, 6.69) | < .001 |
| $\theta_{\mathcal{L}}$ : Surface (Lingual) | 0.93 | 0.34 | (0.27, 1.60) | 0.006 |
| $\theta_{\mathcal{O}}$ : Surface (Occlusal) | -1.01 | 0.29 | (-1.59, -0.44) | < .001 |
| $\theta_{\mathcal{A}}$ : Side (Anterior) | -0.20 | 0.37 | (-0.93, 0.52) | 0.583 |
| $\theta_{\mathcal{G}}$ : Side (Left) | -0.38 | 0.27 | (-0.91, 0.14) | 0.151 |
| $\theta_{\mathcal{M}}$ : Jaw (Maxillary) | 0.59 | 0.24 | (0.11, 1.07) | 0.016 |
|  |  |  |  |  |

**Table A2.2.3. Estimated standard deviations of random effects of count submodel for excessive brushing pressure duration on each dental surface.**

| Parameter | Coefficient |
| --- | --- |
| $\sigma_{\gamma}$: SD (Intercept: <Session:Participant>) | 0.27 |
| $\sigma_{\alpha0}$: SD (Intercept: Participant) | 0.38 |
| $\kappa$: Overdispersion parameter | 2.06 |

**Table A2.2.4. Estimated standard deviations of random effects of zero-inflation submodel for excessive brushing pressure duration on each dental surface.**

| Parameter | Coefficient |
| --- | --- |
| $\sigma_{\epsilon}$: SD (Intercept: <Session:Participant>) | 0.69 |
| $\sigma_{\delta}$: SD (Intercept: Participant) | 2.39 |

**Table A2.2.5. Estimated participant-level random effects of count submodel for excessive brushing pressure duration on each dental surface.**

| Participant # | Parameter | Estimate | Std. Error | Pr(>\|z\|) | 2.5 % | 97.5 % |
| --- | --- | --- | --- | --- | --- | --- |
| 1 | $\alpha_{01}$ : (Intercept) | 0.289 | 0.232 | 0.213 | -0.166 | 0.743 |
| 2 | $\alpha_{02}$ : (Intercept) | -0.002 | 0.380 | 0.996 | -0.747 | 0.743 |
| 3 | $\alpha_{03}$ : (Intercept) | -0.002 | 0.380 | 0.996 | -0.747 | 0.743 |
| 4 | $\alpha_{04}$ : (Intercept) | -0.002 | 0.380 | 0.996 | -0.747 | 0.743 |
| 5 | $\alpha_{05}$ : (Intercept) | -0.158 | 0.302 | 0.600 | -0.750 | 0.434 |
| 6 | $\alpha_{06}$ : (Intercept) | -0.599 | 0.349 | 0.086 | -1.283 | 0.084 |
| 7 | $\alpha_{07}$ : (Intercept) | -0.002 | 0.380 | 0.996 | -0.747 | 0.743 |
| 8 | $\alpha_{08}$ : (Intercept) | 0.206 | 0.346 | 0.552 | -0.473 | 0.885 |
| 9 | $\alpha_{09}$ : (Intercept) | -0.002 | 0.380 | 0.996 | -0.747 | 0.743 |
| 10 | $\alpha_{010}$ : (Intercept) | -0.080 | 0.254 | 0.753 | -0.578 | 0.418 |
| 11 | $\alpha_{011}$ : (Intercept) | 0.277 | 0.254 | 0.276 | -0.221 | 0.775 |
| 12 | $\alpha_{012}$ : (Intercept) | -0.022 | 0.324 | 0.947 | -0.656 | 0.613 |

**Table A2.2.6. Estimated participant-level random effects of zero-inflated submodel for excessive brushing pressure duration on each dental surface.**

| Participant # | Parameter | Estimate | Std. Error | Pr(>\|z\|) | 2.5 % | 97.5 % |
| --- | --- | --- | --- | --- | --- | --- |
| 1 | $\delta_{1}$ : (Intercept) | -3.737 | 0.938 | <0.001 | -5.576 | -1.898 |
| 2 | $\delta_{2}$ : (Intercept) | 1.594 | 1.576 | 0.312 | -1.494 | 4.682 |
| 3 | $\delta_{3}$ : (Intercept) | 1.594 | 1.576 | 0.312 | -1.494 | 4.682 |
| 4 | $\delta_{4}$ : (Intercept) | 1.594 | 1.576 | 0.312 | -1.494 | 4.682 |
| 5 | $\delta_{5}$ : (Intercept) | -1.269 | 1.002 | 0.205 | -3.233 | 0.695 |
| 6 | $\delta_{6}$ : (Intercept) | -2.172 | 0.963 | 0.024 | -4.060 | -0.284 |
| 7 | $\delta_{7}$ : (Intercept) | 1.594 | 1.576 | 0.312 | -1.494 | 4.682 |
| 8 | $\delta_{8}$ : (Intercept) | -0.329 | 1.089 | 0.763 | -2.463 | 1.805 |
| 9 | $\delta_{9}$ : (Intercept) | 1.594 | 1.576 | 0.312 | -1.494 | 4.682 |
| 10 | $\delta_{10}$ : (Intercept) | -2.207 | 0.956 | 0.021 | -4.082 | -0.333 |
| 11 | $\delta_{11}$ : (Intercept) | -2.842 | 0.944 | 0.003 | -4.693 | -0.992 |
| 12 | $\delta_{12}$ : (Intercept) | -0.717 | 1.044 | 0.492 | -2.762 | 1.328 |

Also, the estimated value for overdispersion parameter $\eta$ was 0.72.

# A3. Total active brushing duration of each brushing session

## A3.1. Modeling

We fit a negative binomial regression model for the total active duration of each brushing session (measured in counts of 25 Hz samples), with a log-link and random intercepts by participant ID. We also included a participant-specific overdispersion parameters to account for participant-to-participant differences in residual variance.

$$Y_{ij}\sim NB\left( \lambda_{ij},\kappa_{i} \right)$$

Hence:

$$\lambda_{ij}\mathbb{=E}\left( Y_{ij} \right)$$

$$\mathrm{Var} \left( Y_{ij} \right)=\lambda_{ij}\left( 1+\frac{\lambda_{ij}}{\kappa_{i}} \right)$$

We model $\lambda_{ij}$ as follows:

$$log\left( \lambda_{ij} \right)=\beta_{0i}$$

$$\beta_{0i}=\beta_{0}+\alpha_{0i}$$

$$\alpha_{0i}\sim N\left( 0,\sigma_{\alpha_{0}}^{2} \right)$$

$$\log(\kappa_{i})= \eta_{i}$$

The AIC for this model was 1,802.112, which was 8,098.558 less than a zero-inflated Poisson model with the same fixed and random effects. The BIC for this model was 1,841.137, which was 8,065.108 less than the zero-inflated Poisson model with the same fixed and random effects.

## A3.2. Parameter Estimates

The estimated parameters are summarized in the tables below.

**Table A3.2.1. Estimated fixed effects of total active brushing in each brushing session.**

| Parameter | Log-Mean | SE | 95% CI | p |
| --- | --- | --- | --- | --- |
| $\beta_{0}$ : (Intercept) | 7.71 | 0.04 | (7.63, 7.79) | < .001 |

**Table A3.2.2. Estimated participant-specific overdispersion parameter of total active brushing in each brushing session.**

| Parameter | Coefficient | SE | 95% CI | p |
| --- | --- | --- | --- | --- |
| $\eta_{1}$ : Participant1 | 5.34 | 0.51 | (4.34, 6.34) | < .001 |
| $\eta_{2}$ : Participant2 | 3.95 | 0.49 | (3.00, 4.91) | < .001 |
| $\eta_{3}$ : Participant3 | 4.32 | 0.48 | (3.37, 5.26) | < .001 |
| $\eta_{4}$ : Participant4 | 3.78 | 0.47 | (2.86, 4.71) | < .001 |
| $\eta_{5}$ : Participant5 | 0.97 | 0.52 | (-0.05, 1.99) | 0.064 |
| $\eta_{6}$ : Participant6 | 2.97 | 0.47 | (2.06, 3.89) | < .001 |
| $\eta_{7}$ : Participant7 | 4.10 | 0.48 | (3.17, 5.04) | < .001 |
| $\eta_{8}$ : Participant8 | 3.19 | 0.47 | (2.27, 4.10) | < .001 |
| $\eta_{9}$ : Participant9 | 2.21 | 0.46 | (1.30, 3.12) | < .001 |
| $\eta_{10}$ : Participant10 | 5.24 | 0.51 | (4.24, 6.23) | < .001 |
| $\eta_{11}$ : Participant11 | 3.68 | 0.47 | (2.76, 4.60) | < .001 |
| $\eta_{12}$ : Participant12 | 2.86 | 0.46 | (1.95, 3.77) | < .001 |

**Table A3.2.3. Estimated random effect standard deviation of total active brushing in each brushing session.**

| Parameter | Coefficient |
| --- | --- |
| $\sigma_{\alpha_{0}}$ : SD (Intercept: Participant) | 0.12 |

**Table A3.2.4. Estimated participant-level random effects of total active brushing per brushing session.**

| Participant # | Parameter | Estimate | Std. Error | Pr(>\|z\|) | 2.5 % | 97.5 % |
| --- | --- | --- | --- | --- | --- | --- |
| 1 | $\alpha_{01}$ : (Intercept) | 0.172 | 0.048 | <0.001 | 0.078 | 0.266 |
| 2 | $\alpha_{02}$ : (Intercept) | 0.185 | 0.061 | 0.003 | 0.065 | 0.305 |
| 3 | $\alpha_{03}$ : (Intercept) | -0.021 | 0.052 | 0.684 | -0.123 | 0.081 |
| 4 | $\alpha_{04}$ : (Intercept) | 0.045 | 0.059 | 0.438 | -0.069 | 0.160 |
| 5 | $\alpha_{05}$ : (Intercept) | -0.145 | 0.154 | 0.346 | -0.446 | 0.156 |
| 6 | $\alpha_{06}$ : (Intercept) | -0.085 | 0.069 | 0.222 | -0.220 | 0.051 |
| 7 | $\alpha_{07}$ : (Intercept) | -0.028 | 0.054 | 0.604 | -0.133 | 0.078 |
| 8 | $\alpha_{08}$ : (Intercept) | -0.064 | 0.065 | 0.322 | -0.192 | 0.063 |
| 9 | $\alpha_{09}$ : (Intercept) | -0.095 | 0.088 | 0.277 | -0.267 | 0.076 |
| 10 | $\alpha_{010}$ : (Intercept) | 0.124 | 0.048 | 0.010 | 0.030 | 0.219 |
| 11 | $\alpha_{011}$ : (Intercept) | -0.029 | 0.058 | 0.617 | -0.143 | 0.085 |
| 12 | $\alpha_{012}$ : (Intercept) | -0.071 | 0.071 | 0.318 | -0.210 | 0.068 |

To find out the between- and within-person variabilities in active brushing duration, we have summarized the mean and standard deviations estimated from the model and calculated empirically from the samples in the table below:

**Table A3.2.5. Participant-specific estimates of active brushing duration.**

| Participant | sample mean (seconds) | sample var (seconds^2) | sample sd (seconds) | lambda (samples) | lambda (seconds) | kappa | model var (seconds^2) | model sd (seconds) |
| --- | --- | --- | --- | --- | --- | --- | --- | --- |
| 1 | 107 | 58.2 | 7.63 | 2646 | 106 | 209 | 57.8 | 7.6 |
| 2 | 110 | 195 | 14 | 2680 | 107 | 52.2 | 224 | 15 |
| 3 | 87 | 103 | 10.1 | 2180 | 87.2 | 75.1 | 105 | 10.2 |
| 4 | 93.9 | 171 | 13.1 | 2330 | 93.2 | 44 | 201 | 14.2 |
| 5 | 46.7 | 477 | 21.8 | 1927 | 77.1 | 2.63 | 2263 | 47.6 |
| 6 | 79.3 | 292 | 17.1 | 2046 | 81.9 | 19.6 | 345 | 18.6 |
| 7 | 86.3 | 113 | 10.6 | 2166 | 86.6 | 60.6 | 127 | 11.3 |
| 8 | 81.9 | 287 | 16.9 | 2088 | 83.5 | 24.2 | 291 | 17.1 |
| 9 | 74.9 | 486 | 22 | 2025 | 81 | 9.15 | 720 | 26.8 |
| 10 | 101 | 59.9 | 7.74 | 2522 | 101 | 188 | 58.2 | 7.63 |
| 11 | 86.1 | 192 | 13.8 | 2163 | 86.5 | 39.6 | 192 | 13.9 |
| 12 | 80.5 | 368 | 19.2 | 2074 | 83 | 17.4 | 399 | 20 |

# As it can be seen the empirical values and the model estimates are close. We report the between- and within- person variability in brushing duration to be 16.69 and 14.50 seconds by calculating the standard deviation of the second column and the mean of the fourth column in Table A3.2.5., respectively. By dividing the calculated between- and within- person variability to the mean of the second column, we can calculate the coefficients of between- and within- person variability to be 0.19 and 0.17 which is more than 5%; hence we consider them substantial.
