# Supplementary material for: A naturalistic study of brushing patterns using powered toothbrushes
Source: PLoS One. 2022 May 19;17(5):e0263638. doi: 10.1371/journal.pone.0263638 (PMC9119504; doi:10.1371/journal.pone.0263638)
Supplement: Attachment — Submitted filename: Response to reviewers.docx [file pone.0263638.s003.docx]

A naturalistic study of brushing patterns using electric toothbrushes

Essalat, Morrison, Kak, Chang, Roig, Kulchar, Padilla, Shetty

[PONE-D-22-02067] - [EMID:48f78bddccdd4d40]

Responses to the reviewers’ comments

Dear Editor,

I am delighted to learn the favorable reaction of the reviewers.

Allow me to clarify the residual issue of redundant sentences (Critique 6- Reviewer 1).

*There are some redundant sentences in the discussion. Specifically, Page 10, 11
Lines 210 – 212 and Page 11 Line 224-225.*

In writing the discussion, we followed the general guidelines provided by PLOS (<https://plos.org/resource/how-to-write-conclusions/>).

The first paragraph of the discussion section provides a high-level summary of our main findings. The subsequent paragraphs begin with a brief description of each finding, followed by a brief interpretation of the finding and a comparison/contrast with findings by other investigators.
